# A Case of Lung Adenocarcinoma Originating from an Old Posttraumatic Scar in a Young Patient

**DOI:** 10.1155/2019/8395389

**Published:** 2019-04-08

**Authors:** Fatima Ahmed, Hassaan Yasin, Hesham E. Mohamed

**Affiliations:** ^1^Department of Internal Medicine, Charleston Area Medical Center-West Virginia University, 3200 Maccorkle Avenue SE, Charleston, WV 25304, USA; ^2^Department of Pulmonary Disease and Critical Care Medicine, Charleston Area Medical Center-West Virginia University, 3200 Maccorkle Avenue SE, Charleston, WV 25304, USA

## Abstract

A relationship between lung scarring and cancer has been recognized for many decades but more evidence is needed to strengthen this association. A 34-year-old nonsmoker male with a history of left lower lobe lung scar secondary to a pulmonary contusion from a motor vehicle accident in 2012 was admitted with shortness of breath and cough. A computed tomography (CT) angiography of the chest demonstrated bilateral pulmonary emboli, left lower lobe mass, left lung septal thickening, and mediastinal lymphadenopathy. A CT-guided biopsy of the mass was performed, and pathology was consistent with lung adenocarcinoma. Staging work-up revealed a widely metastatic disease. The patient developed severe complications requiring hospitalization after the first cycle of chemotherapy and subsequently passed away. Lung scar carcinoma originates around peripheral scars resulting from a variety of infections, injuries, and lung diseases. It has poor prognosis because it metastasizes from relatively small lesions. Our case further endorses that lung scarring can potentially lead to the development of cancer. Furthermore, we want to highlight the need to conduct studies to determine if monitoring this patient population with periodic imaging can have a survival benefit.

## 1. Introduction

Lung cancer is the leading cause of cancer-related mortality worldwide. Although lung cancer is predominantly seen in smokers, never-smokers (individuals who have smoked less than 100 cigarettes in their lifetime) account for 20% of cases globally [1]. Adenocarcinoma is the most common histologic type among both groups [2]. Although cigarette smoking is, by far, the biggest risk factor for developing lung cancer, age, occupational exposures, environmental pollution, race, gender, and preexisting lung disease are all important contributors [3]. There is an etiologic relationship between lung scarring and the development of pulmonary carcinoma [4]. In this article, we present a case of lung adenocarcinoma that originated from a posttraumatic scar.

## 2. Case Presentation

**Figure 1:**
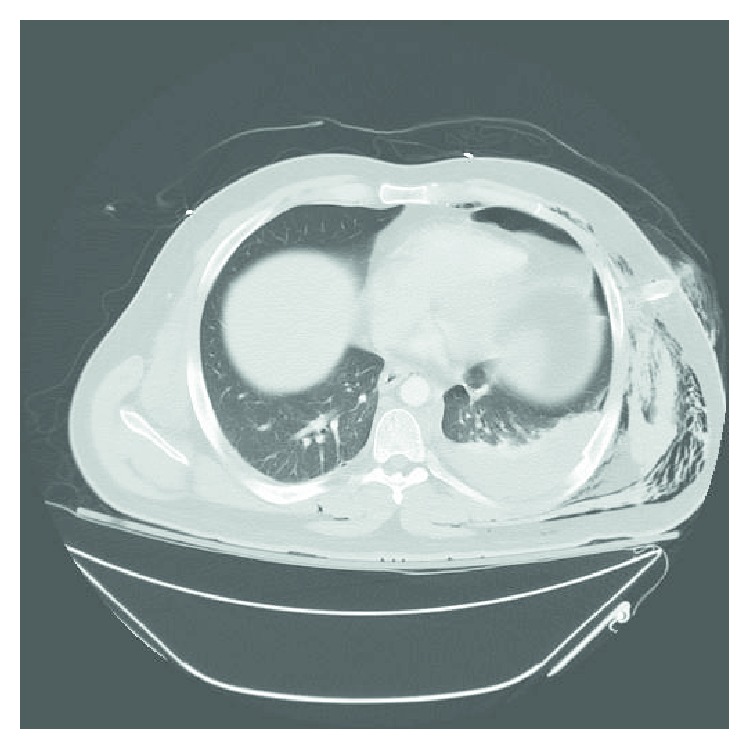
Computed tomography of the chest from January 2012 showing left-sided hemothorax and subcutaneous emphysema.

**Figure 2:**
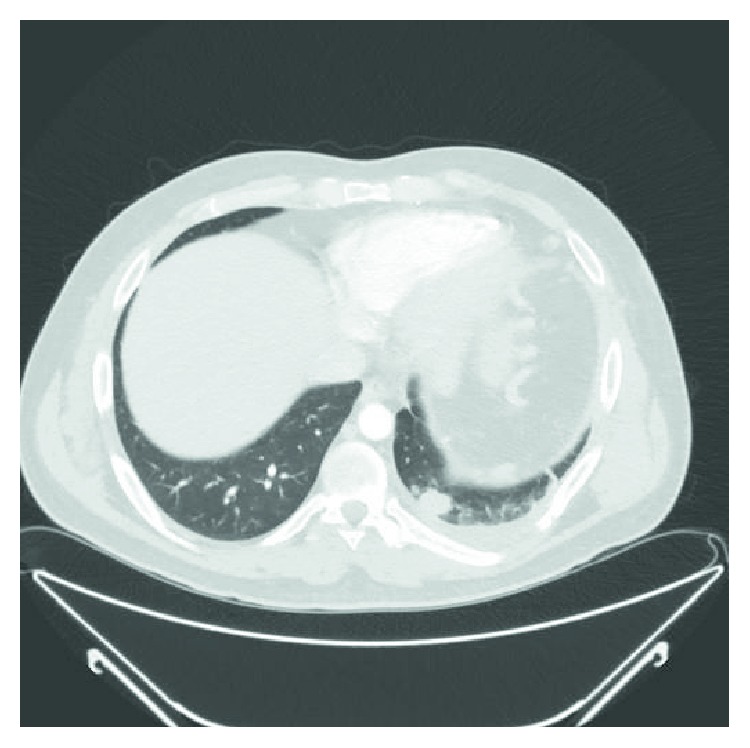
Computed tomography of the chest from May 2012 showing a left lower lobe residual nodular density.

A 34-year-old nonsmoker male patient presented to the emergency room with a one-week history of dyspnea, pleuritic chest pain, and a nonproductive cough. His past medical history was significant for a motor vehicle accident five years earlier that had resulted in multiple left-sided rib fractures, pulmonary contusions, and a hemopneumothorax requiring tube thoracostomy (Figure 1); this left a residual nodular density in the left lower lobe (Figure 2). On physical exam, he was afebrile, normotensive, tachycardic, hypoxic and in mild respiratory distress and had diminished breath sounds bilaterally.

**Figure 3:**
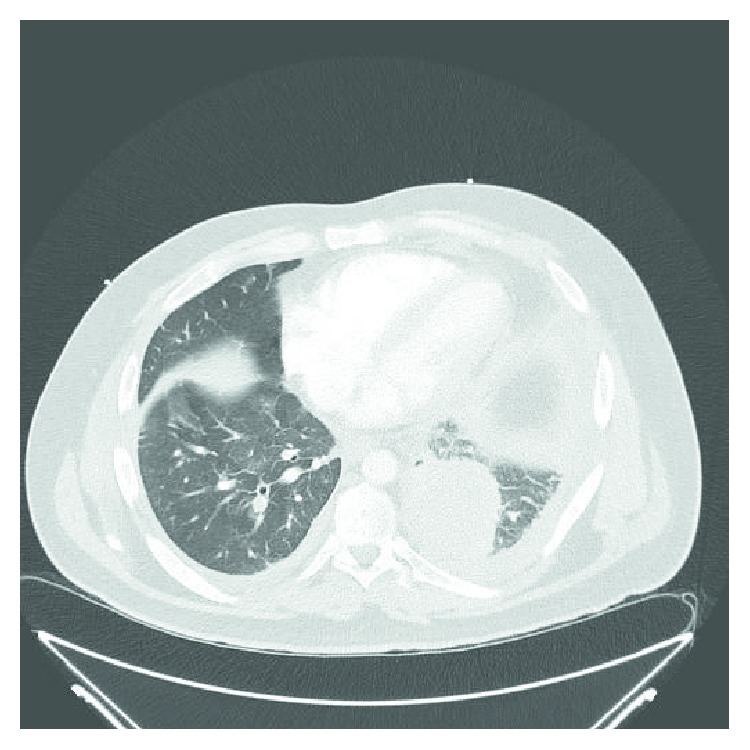
Computed tomography of the chest from June 2017 showing a left lower lobe opacity with preseptal thickening and a small pleural effusion.

Laboratory work-up showed a white blood cell count of 20,500/mm^3^. His electrocardiogram showed sinus tachycardia. X-ray imaging of the chest revealed a left lung base opacification. Computed tomographic (CT) angiography of the lung demonstrated bilateral pulmonary emboli, a 6.6 × 5.4 cm opacity in the left lower lobe with interlobular septal thickening, prominent interstitial infiltrates within the left lung, and paratracheal lymphadenopathy (Figure 3). This opacity had enlarged significantly when compared to the one visualized at the same location in 2012 (Figure 2). The patient was treated with IV heparin for pulmonary embolism. A CT-guided biopsy of the lung mass and endobronchial ultrasonographic sampling of the mediastinal lymph nodes established the diagnosis of lung adenocarcinoma. Further imaging obtained to complete the staging work-up revealed widespread metastasis to the bone.

Immunohistochemical testing for programmed death-ligand 1 showed 50 percent expression. Molecular analysis did not show the presence of EGFR mutations and ALK/ROS1 translocations. While these tests were pending, treatment with carboplatin and paclitaxel was started. However, after the first cycle of chemotherapy, the patient became critically ill and was hospitalized. Subsequently, he developed features of disseminated intravascular coagulation and passed away shortly thereafter.

## 3. Discussion

Lung scar carcinoma (LSC) was first described in 1939 by Friedrich as a form of lung cancer that originates from peripheral scars in the lung. These, in turn, may arise from infection, injury, intrinsic pulmonary disease, or recurrent episodes of tumor necrosis and healing [4]. The most common etiologic factor for the development of LSC is scarring secondary to tuberculosis, but it is also known to occur in the setting of pneumonia, pulmonary abscess, bronchiectasis, and pulmonary infarction [5]. The pathogenesis involves production of acute-phase reactants during the inflammatory response, which leads to recruitment of leukocytes. These activated cells produce reactive oxygen species (ROS) that mediate mutagenic changes in deoxyribonucleic acid (DNA) and damage proteins involved in the maintenance of genomic stability [6, 7]. Chronic inflammation promotes persistent DNA damage and eventual activation of oncogenes with subsequent neoplastic transformation. Inflammatory mediators such as tumor necrosis factor (TNF), transforming growth factor (TGF), and interleukins 1, 4, 6, and 13 cause angiogenesis and fibrosis [6, 8] and are also implicated in tumorigenesis. Notably, the Prostate, Lung, Colorectal, and Ovarian (PLCO) Cancer Screening Trial has demonstrated a two-fold increased risk of lung cancer associated with the detection of pulmonary scarring on chest X-ray [9].

LSC is most commonly subpleural adenocarcinoma with no evidence of bronchial origin and is characterized histologically by contiguity with dense, hyalinized scar tissue that itself does not comprise any tumor cells [4]. It is hypothesized that impedance of lymphatic drainage by scar tissue can lead to accumulation of malignant cells with subsequent accelerated vascular and/or lymphatic spread of LSC, as was observed in our patient [10]. LSC therefore carries a dismal prognosis, with a five-year survival rate of only 5%, as opposed to 22% for non-LSC adenocarcinoma and 28% for non-LSC adenosquamous cell carcinoma [11]. It is, therefore, important to diagnose and treat this disease early. Because of the physical overlap that exists between LSC and scar tissue on CT imaging, the former is often misdiagnosed as an old fibrotic lesion in the absence of sequential imaging, particularly in younger patients. However, radiographic changes in scar tissue, such as expansion, vacuolation, vessel convergence within the region of fibrosis, and emergence of well-defined surrounding ground-glass opacities and spiculations, strongly support the presence of LSC. Vacuolation and ground-glass opacities are especially useful in differentiating malignant from benign lesions. Ground-glass opacities surrounding areas of scar tissue are considered to be reliable markers of early-stage LSC but tend to consolidate over time and so run the risk of being missed if not detected early enough [12].

## 4. Conclusion

Our case serves to highlight the link between pulmonary scarring and carcinogenesis, as well as to demonstrate the relentless course of lung scar carcinoma, which necessitates timely diagnosis. The USPSTF recommends annual screening for lung cancer with a low-dose CT scan of chest in adults aged 55 to 80 who have a 30 pack-year smoking history. However, no such recommendations exist for individuals with a known history of lung scarring. Our case reinforces the need for periodic CT imaging of the chest in this group of individuals who are at increased risk of this form of malignancy and underscores the need for evidence to determine the survival benefit of such a measure.
